# [Retracted] Upregulation of GRIM-19 suppresses the growth of oral squamous cell carcinoma *in vitro* and *in vivo*

**DOI:** 10.3892/or.2025.8955

**Published:** 2025-07-21

**Authors:** Minghe Li, Zhihong Li, Chongyang Liang, Chengmin Han, Wei Huang, Fei Sun

Oncol Rep 32: 2183–2190, 2014; DOI: 10.3892/or.2014.3423

Following the publication of this paper, it was drawn to the Editor's attention by a concerned reader that certain of the western blot data shown in Figs. 1C and 5C on p. 2186 and p. 2188 respectively were strikingly similar to data that had already been published in articles written by different authors at different research institutes, or which had been included in articles that were already under consideration for publication.

Upon investigating this matter independently in the Editorial Office, we were able to confirm the reader's concerns. Therefore, due to the fact that the contentious western blot data in the above article had already been published prior to its submission to *Oncology Reports*, the Editor has decided that this paper should be retracted from the Journal. The authors were asked for an explanation to account for these concerns, but the Editorial Office did not receive a reply. The Editor apologizes to the readership for any inconvenience caused.

